# Liver- and Spleen-Specific Immune Responses in Experimental *Leishmania martiniquensis* Infection in BALB/c Mice

**DOI:** 10.3389/fvets.2021.794024

**Published:** 2021-12-17

**Authors:** Woraporn Sukhumavasi, Theerayuth Kaewamatawong, Nawaphat Somboonpoonpol, Montakan Jiratanh, Juntra Wattanamethanont, Morakot Kaewthamasorn, Saovanee Leelayoova, Saruda Tiwananthagorn

**Affiliations:** ^1^Parasitology Unit, Department of Pathology, Faculty of Veterinary Science, Chulalongkorn University, Bangkok, Thailand; ^2^Feline Infectious Disease and Health for Excellence Research Unit, Microbial Food Safety and Antimicrobial Resistance Research Unit, Animal Vector-Borne Disease Research Unit, Chulalongkorn University, Bangkok, Thailand; ^3^Veterinary Pathology Unit, Department of Veterinary Pathology, Faculty of Veterinary Science, Chulalongkorn University, Bangkok, Thailand; ^4^Parasitology Section, National Institute of Animal Health, Department of Livestock Development, Ministry of Agriculture and Cooperatives, Bangkok, Thailand; ^5^Veterinary Parasitology Research Unit, Department of Pathology, Faculty of Veterinary Science, Chulalongkorn University, Bangkok, Thailand; ^6^Department of Parasitology, Phramongkutklao College of Medicine, Bangkok, Thailand; ^7^Department of Veterinary Biosciences and Veterinary Public Health, Faculty of Veterinary Medicine, Chiang Mai University, Chiang Mai, Thailand; ^8^Research Center of Producing and Development of Products and Innovations for Animal Health and Production, Faculty of Veterinary Medicine, Chiang Mai University, Chiang Mai, Thailand

**Keywords:** *Leishmania martiniquensis*, BALB/c mouse, hepatic granuloma, parasite persistence, *iNOS*, *IL-10*, *TNF-*α, *IFN-*γ

## Abstract

*Leishmania martiniquensis* is a neglected cause of an emerging leishmaniasis in many countries, including France, Germany, Switzerland, the United States of America, Myanmar, and Thailand, with different clinical manifestations ranging from asymptomatic, cutaneous (CL), visceral (VL), and atypically disseminated CL and VL. The persistence of parasites and the recurrence of the disease after treatment are challenges in controlling the disease. To explore efficient prophylaxis and therapy, this study aimed to investigate infection outcome and organ-specific immune responses after inoculation with *L. martiniquensis* (MHOM/TH/2011/PG; 5 x 10^6^ promastigotes) in BALB/c mice *via* intravenous and intraperitoneal routes. A quantitative PCR technique, targeting *L. martiniquensis* ITS1, was primarily established to estimate the parasite burden. We found that the infection in the liver resolved; however, persistent infection was observed in the spleen. Histopathology with *Leishmania*-specific immunostaining revealed efficient hepatic granuloma formation, while splenic disorganization with parasitized macrophages at different locations was demonstrated. The mRNA expression of Th1 cytokines (*IFN-*γ*, TNF-*α*, IL-12p40*) and *iNOS* in the liver and spleen was upregulated. In addition, high expression of *IL-10* was observed in the spleen in the chronic phase, revealing a significant moderate correlation with the parasite persistence [r_(12)_ = 0.72, *P* = 0.009]. Further clarification of the mechanisms of persistent infection and experimental infection in immunosuppressed murine models are warranted.

## Introduction

Leishmaniasis is a vector-borne disease caused by several species of intracellular protozoa belonging to the *Leishmania* genus. The main clinical manifestations, including cutaneous leishmaniasis (CL), mucocutaneous leishmaniasis (MCL), and visceral leishmaniasis (VL), are generally associated with the *Leishmania* species. Based on the molecular analysis of multiple genes, including internal transcribed ribosomal RNA spacer 1 (*ITS1*), RNA polymerase II (*POLR2*), heat shock protein 70 (*HSP70*), and the ribosomal protein L23a (*RPL23a*), *Leishmania (Mundinia) martiniquensis* and *Leishmania (M.) orientalis*, have been identified as the causative *Leishmania* species in Thailand (1–3).

*Leishmania martiniquensis* is the major species causing autochthonous leishmaniasis in Thailand in immunocompetent and immunosuppressed patients, and has a wide geographical distribution that includes France, Germany, Switzerland, the United States of America, and Myanmar (1). Various clinical manifestations, including asymptomatic, CL alone, VL alone, and atypically disseminated cutaneous and visceral (DCL/VL) concomitant forms, have been reported in cases of *L. martiniquensis* infection, especially in patients with a human immunodeficiency virus (HIV)/acquired immunodeficiency syndrome (AIDS) coinfection (1, 4, 5). The clinical characteristics of VL caused by *L. martiniquensis* are comparable with typical VL reported in *L. donovani* and *L. infantum* infections, including prolonged fever, anemia, hepatosplenomegaly, and cachexia. All documented VL cases caused by *L. martiniquensis* infection present a chronic infection and are occasionally asymptomatic in human and animal reservoirs (1).

Loss of control over parasite persistence in VL causes reactivation of the infection that is often observed in immunocompromised patients. Furthermore, recurrence after treatment is a challenge in controlling *Leishmania* infection (6, 7). To develop an effective prophylaxis, vaccine or therapeutic regimens against VL, a better understanding of the precise pathology of the disease and the immune response is required. Various experimental VL studies have been conducted in mouse models to elucidate the pathogenesis, disease progression, and drugs for treatment, and to describe organ-specific immunity (8–10). The outcome of the disease in the mouse model is influenced by several experimental parameters, such as the genetic background of the mouse, the strain and genotype of the parasite, the inoculation route/infection site, the dose of the parasite and the presence of saliva from phlebotomine (11, 12).

BALB/c mice are the most widely used experimental animal model for the study of immunopathological changes during VL, as their clinical features resemble those in human VL; for example, hepatosplenomegaly or the disruption of splenic tissues (11). Few experimental studies of *L. martiniquensis* employing BALB/c mice have been reported (13, 14). Garin et al. (13) demonstrated the differences in parasite growth and dissemination in the lymph nodes, liver, spleen, and brain of BALB/c mice *via* subcutaneous or intravenous infection with two strains of *L. martiniquensis* (15), isolated from a patient with HIV infection (MHOM/MQ/92/MARl) and from an immunocompetent patient (MHOM/MQ/97/MAR2). An infection resolving in the liver after intravenous infection with both *Leishmania* strains was reported, although infection with MHOM/MQ/92/MARl revealed clearance of the latter parasite. In another study, Intakhan et al. (14) explored clinical progression, parasitic load, and histological alterations of the liver and spleen using a different strain of *L. martiniquensis* (MHOM/TH/2013/LSCM3) in BALB/c mice and Syrian golden hamsters by intraperitoneal inoculation.

To treat and control *L. martiniquensis* infection more efficiently, the mechanisms of host defense need to be thoroughly explored. To accomplish this aim, we investigated organ-specific immune responses, including the liver and spleen, after *L. martiniquensis* infection (strain MHOM/TH/2011/PG) using inbred BALB/c mice, and compared the outcomes between intravenous and intraperitoneal routes of inoculation. The dynamics of parasite burden, histopathological changes, and cytokine mRNA expression levels in the acute and chronic phases of infection were explored.

## Materials and Methods

### Experimental Animals

Female-specific pathogen-free BALB/c mice, 6–8 weeks of age, were purchased from the National Laboratory Animal Center, Mahidol University. Animals were acclimatized for 3 weeks and then maintained in the Animal Biosafety Level 2 facility at the National Institute of Animal Health (NIAH). The experimental protocols were reviewed and approved by the NIAH animal use committee [EA-001/57(R)].

### Preparation for *L. martiniquensis*

*Leishmania martiniquensis* [strain MHOM/TH/2011/PG; Zymodeme MON-229 (1, 16)] was maintained by passage of the frozen stabilized parasites in liquid Schneider's *Drosophila* medium with L-glutamate (Sigma-Aldrich, MO, USA), supplemented with 20% heat-inactivated fetal bovine serum (Merck Millipore, Darmstadt, Germany), 100 U/mL of penicillin, 100 μg/mL of streptomycin, and 50 μg/mL of gentamicin at 25°C. The stationary growth phase of the subcultures with less than five passages was used for mouse inoculation. The stationary phase promastigotes were washed twice and resuspended in 1X phosphate buffered saline to a final concentration of 5 ×10^6^ promastigotes in 200 μL.

### Experimental Infection and Sample Collection

A total of 40 mice were divided into 3 groups: intraperitoneal infection (IP, 16 mice), intravenous infection (IV, 16 mice), and uninfected control (CTRL, 8 mice). Mice in the treatment groups were inoculated with 5 × 10^6^ promastigotes of *L. martiniquensis* each. Mice in the intravenous route group were inoculated *via* the lateral tail vein. On 7-, 14-, 28-, and 112-days post-infection (dpi), four inoculated mice from each inoculation group and 2 CTRL mice were euthanized consecutively. A necropsy was performed to collect whole blood samples (*via* cardiac puncture), liver, and spleen. Organ weighing was conducted, and each organ was divided into three sections for several purposes. Two sections were preserved in RNA*later*™ Stabilization Solution (Invitrogen, Vilnius, Lithuania) for genomic DNA and mRNA extraction, as well as further parasite burden quantification and cytokine mRNA level expression. The last section was fixed in 10% neutral phosphate-buffered formalin for histopathological examination.

### Determination of Parasite Burdens

Genomic DNA (gDNA) was isolated from the liver and spleen, using the QIAamp^®^ DNA Mini Kit (Qiagen, MA, USA). Quantitative real-time PCR (qPCR) was established to quantify the relative amount of *L. martiniquensis* DNA with respect to mouse glyceraldehyde-3-phosphate dehydrogenase (m*GAPDH*) DNA (17, 18). In the present study, an original oligonucleotide primer set, targeting the *Leishmania* ITS1 region (*Leishmania-*ITS1), was designed using Primer3Plus (19), (L.ITS1.PCM2.4.6-qF and L.ITS1.PCM2.4.6-qR in Table 1).

**Table 1:** Target genes and primers for qPCR and RT-qPCR used in this study.

**Genes**	**Name**	**Primer sequences (5′−3′)**	**Amplicon size (bp)**	**References**
*Leishmania-*ITS1	L.ITS1.PCM2.4.6-qF	CTGGATCATTTTCCGATGATTACA	300	This study
	L.ITS1.PCM2.4.6-qR	CACGTTATGTGAGCCGTTATC		
Mouse *GAPDH*	mGAPDH-qF	TCACCACCATGGAGAAGGC	168	(17)
	mGAPDH-qR	GCTAAGCAGTTGGTGGTGCA		
Mouse *IFN-γ*	mIFNg-qF	TCAAGTGGCATAGATGTGGAAGAA	92	(18, 28)
	mIFNg-qR	TGGCTCTGCAGGATTTTCATG		
Mouse *TNF-α*	mTNFa-qF	CATCTTCTCAAAATTCGAGTGACAA	175	
	mTNFa-qR	TGGGAGTAGACAAGGTACAACCC		
Mouse *IL-12p40*	mIL12p40-qF	GGAAGCACGGCAGCAGAATA	180	
	mIL12p40-qR	AACTTGAGGGAGAAGTAGGAATGG		
Mouse *IL-2*	mIL2-qF	CCTGAGCAGGATGGAGAATTACA	141	
	mIL2-qR	TCCAGAACATGCCGCAGAG		
Mouse *IL-4*	mIL4-qF	ACAGGAGAAGGGACGCCAT	95	
	mIL4-qR	GAAGCCCTACAGACGAGCTCA		
Mouse *IL-10*	mIL10-qF	GGTTGCCAAGCCTTATCGGA	191	
	mIL10-qR	ACCTGCTCCACTGCCTTGCT		
Mouse *iNOS*	mINOS-F	CAGCTGGGCTGTACAAACCTT	95	
	mINOS-R	CATTGGAAGTGAAGCGTTTCG		

The qPCR was performed using the Applied Biosystems 7300 Real Time PCR system (Applied Biosystems, CA, USA), following the manufacturer's instructions. A typical 20-μL reaction mixture contained approximately 100 ng of genomic DNA, the SensiFAST™ SYBR Hi-ROX Kit (Bioline, MA, USA), and 0.4 μM of each primer (Table 1). All samples were run in triplicate and underwent an initial 3-min incubation step at 95°C, followed by 40 cycles of 5 s at 95°C and 30 s at 60°C. The final extension was incubated for 30 s at 60°C for dissociation and measurement of the melting temperature (Tm) of the qPCR products. The average threshold cycle (Ct) of amplification values was determined, and the standard deviation (SD) of all reactions was analyzed by the software provided with the instrument. The relative amounts of *Leishmania-*ITS1 to the housekeeping m*GAPDH* gene were then calculated using a standard curve with the efficiency correction method, according to Livak et al. (20), and reported as the proportion of quantity of these two genes.

The standard curves of the two genes were primarily established with a two-fold serial dilution of DNA template concentrations (0.3125 to 10 ng). To test the specificity of the newly established primers, the cross-amplification of the assay with other viscerotropic *Leishmania* species was determined, using the gDNA of the cultured *L. orientalis* (MHOM/TH/2010/TR, formerly *L. siamensis*)*, L. infantum* (MCAN/TR/2000/EP55), and *L. donovani* (MHOM/SU/62/2S-25M-C2) (21) as the DNA template of reaction.

### Histopathological and Immunohistochemical Analyses

Livers and spleens were fixed in 10% neutral phosphate buffered formalin. The paraffin-embedded organs were cut into 4 μm-thick sections, followed by hematoxylin and eosin staining for light microscopy. For the detection of parasites, liver sections were performed by indirect immunostaining using human serum infected with *L. martiniquensis* (1) (1:500 dilution) and peroxidase-conjugated AffiniPure goat anti-human IgG heavy and light chain antibody (1:500 dilution; Jackson ImmunoResearch, PA, USA). The peroxidase activity was visualized using a solution of 3,3'-diaminobenzidine (DAB) (Wako, Tokyo, Japan) and H_2_O_2_ (pH 7.0) for 4 min. The sections were washed in distilled water and counterstained with Mayer's hematoxylin before dehydration and mounting.

The cell-mediated immune response (CMIR) of the liver against parasitized Kupffer cells was classified into no granuloma, immature granuloma, mature granuloma, and involuting granuloma (22, 23). The number of each response was counted in 25 consecutive microscopic fields per mouse at ×400 magnification. The histopathological reaction and CMIR of the spleen were determined, according to previously published protocols (24–27).

### Measurement of Cytokine mRNA Levels in the Liver and Spleen

Quantitative reverse-transcriptase polymerase chain reaction (RT-qPCR) was used to examine the expression of cytokines in the liver and spleen, including interferon-gamma *(IFN-*γ*)*, tumor necrosis factor-alpha *(TNF-*α*)*, interleukin 12 subunit 40 *(IL-12p40), IL-2, IL-4, IL-10*, and inducible nitric oxide synthase *(iNOS)*. Total RNA was extracted from the liver using the TRIzol^®^ reagent (Invitrogen, CA, USA). Approximately 500 ng of RNA was reverse transcribed into cDNA using the SuperScript^TM^ VILO™ cDNA Synthesis Kit (Invitrogen, CA, USA). The qPCR reaction was carried out as described above using cDNA, and the oligonucleotide primers for each cytokine are shown in Table 1 (18, 23, 28). The relative expression levels of cytokine to housekeeping m*GAPDH* in each sample were calculated based on the standard curve and efficiency correction method, and the data were presented in fold changes for the expression level in the naïve mice and the compared data between the different time points at 14, 28, and 112 dpi.

### Statistical Analyses

Statistical analyses between the IP and IV groups, and among the indicated time points were performed using the Student's *t*-test, the two-way ANOVA, and the *post hoc* Bonferroni test (Prism software version 6, GraphPad, CA, USA). Pearson's correlation coefficient was used to determine the relationship between parasite burden and organ weight and between parasite burden and cytokine expression level. Organ weight, parasite burden, hepatic immune response, and cytokine expression levels were presented as the mean values ± standard error (SE), unless otherwise stated, and a *P*-value < 0.05 was considered statistically significant. The CMIR in the spleen was reported as a descriptive analysis.

## Results

### Contrast of Disease Outcomes and Parasite Burden in the Liver and Spleen After *L. martiniquensis* Infection

#### A Developed qPCR Assay for the Quantification of *L. martiniquensis*

**Figure 1:**
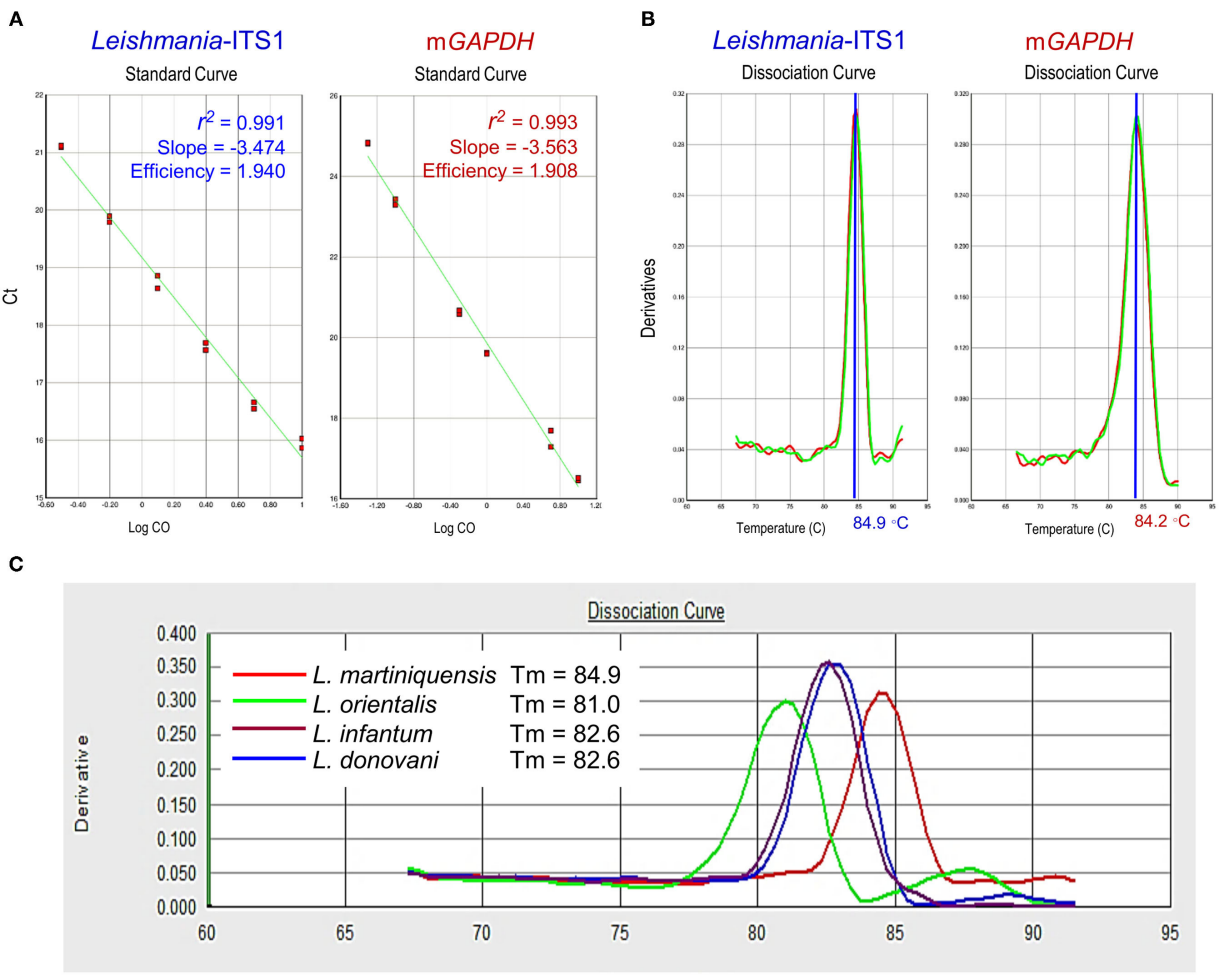
Technical performance of qPCR assays of *Leishmania*-ITS1 and m*GAPDH*. **(A)** Amplification efficiency, correlation coefficients (r^2^), and slope of qPCR of *L. martiniquensis*, using primer L.ITS1.PCM2.4.6-qF and L.ITS1.PCM2.4.6-qR, and m*GAPDH*. Mean Ct values are plotted from triplicates tested against serial dilutions. Each point represents the Ct of an individual sample, with the plot of Ct values and parasite equivalent fitting a linear function. **(B)** Dissociation curve revealing melting temperature (Tm) of *Leishmania*-ITS1 and m*GAPDH* amplicons. **(C)** Specificity of *Leishmania*-ITS1 qPCR to differentiate *L. martiniquensis* from other viscerotropic *Leishmania* species, characterized by the distinct Tm values among *L. martiniquensis, L. orientalis, L. donovani*, and *L. infantum*.

In this study, the newly designed primers, L.ITS1.PCM2.4.6-qF and L.ITS1.PCM2.4.6-qR, were able to amplify *L. martiniquensis gDNA*, which showed a high amplification efficiency of 1.940 (a value of 2 indicates 100% PCR efficiency), with correlation coefficients (*r*^2^) of 0.991 and slopes of −3.47. The standard curve of the m*GAPDH* qPCR assay was also linear, with an *r*^2^ value of 0.993, and slope of −3.56, corresponding to an efficiency of 1.91 (Figure 1A). The Tm of the amplified qPCR products of *L. martiniquensis*-ITS1 and m*GAPDH* were ~84.9 and 84.2°C, respectively (Figure 1B).

Regarding the specificity of the developed *Leishmania*-ITS1 qPCR assay, we found that the primers L.ITS1.PCM2.4.6-qF and L.ITS1.PCM2.4.6-qR could discriminate *L. martiniquensis* from other viscerotropic *Leishmania* species, including *L. infantum, L. donovani* and *L. orientalis*, with different Tm values of the amplified amplicons of each species (Figure 1C). The Tm of the *L. martiniquensis* amplicon was 84.9°C, while that of *L. orientalis* was much lower at 81.0°C, and the Tm values of *L. infantum* and *L. donovani* were similar at 82.6°C. Under the conditions established for each qPCR assay, no amplification of the negative control sample or blank was detected.

#### Slight Hepatomegaly and Remarkable Splenomegaly After Infection With *L. martiniquensis*

**Figure 2:**
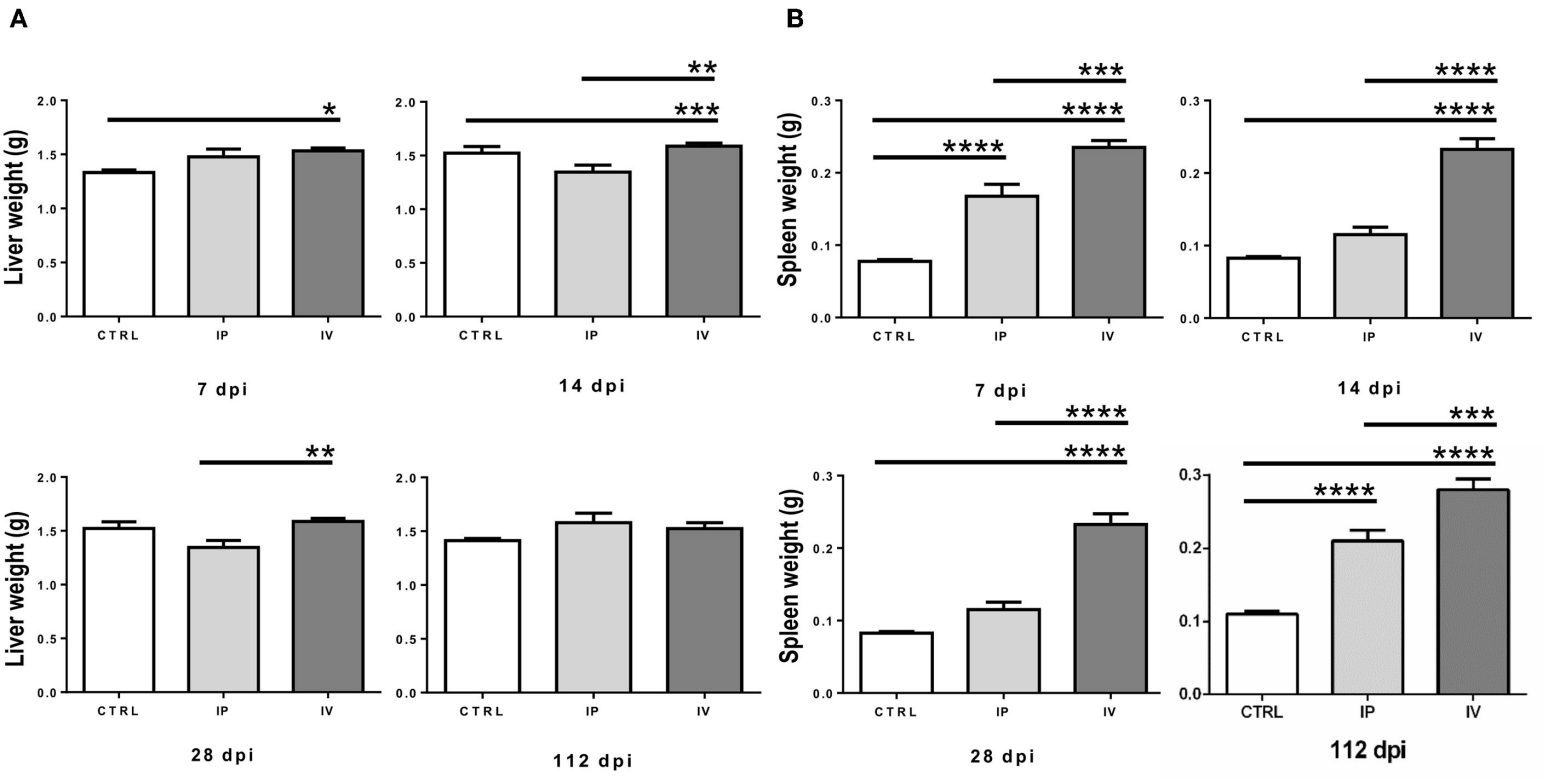
Comparison of liver weight **(A)** and spleen weight **(B)** of BALB/c mice after *Leishmania martiniquensis* infection at 7, 14, 28, and 112 dpi by IV (intravenous) inoculation, IP (intraperitoneal) inoculation, and CTRL group. *P < 0.05; **P < 0.01; ***P < 0.001; ****P < 0.0001.

No apparent clinical signs were observed after *L. martiniquensis* infection in IP or IV-inoculated mice. From necropsy, an enlargement of the liver and spleen was observed in all animals infected by the IP and IV route. A significant modest hepatomegaly was observed in mice in the IV group at 7 dpi and 14 dpi, compared with the CTRL (*P* < 0.05). Compared to IP group, the liver weights of the IV group were larger at 14 dpi and 28 dpi (*P* < 0.01). In the chronic phase of infection, at 112 dpi, there was no significant difference in liver weight between the IV, IP, and CTRL groups (Figure 2A).

In contrast, splenic enlargement in both the IP and IV groups was evident after *L. martiniquensis* infection. The average weights of the spleens in the IV group were significantly higher than the spleens in the IP and CTRL groups throughout the study (*P* < 0.001) (Figure 2B). However, the splenic weights of IP mice were higher than those found in the CTRL group only at 7 dpi and 112 dpi (*P* < 0.0001). No remarkable lesions in the other organs of any control or infected mice were observed.

#### Resolving Infection in the Liver but *L. martiniquensis* Persistence in the Spleen

**Figure 3:**
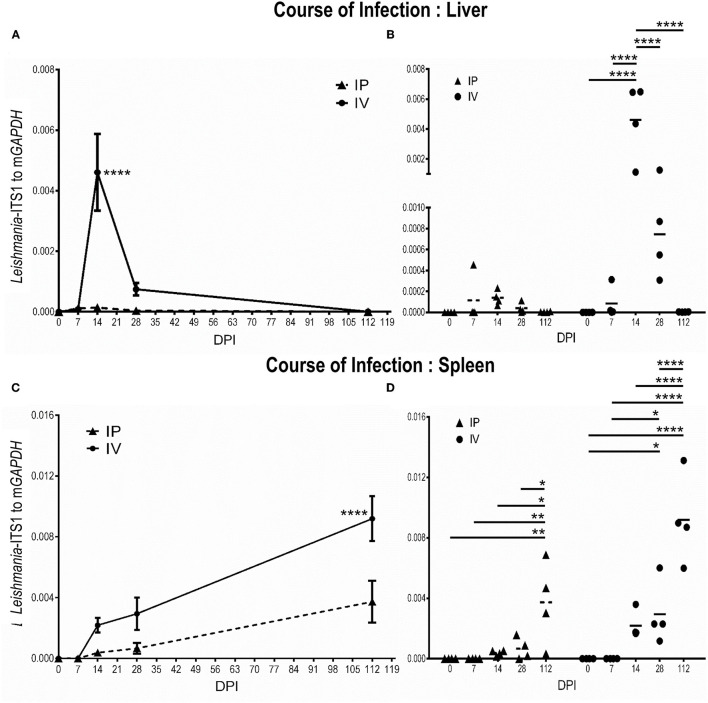
Discrete phases of *Leishmania martiniquensis* infection in the liver **(A,B)** and spleen **(C,D)** of BALB/c mice after inoculation by intraperitoneal (IP) and intravenous routes (IV). At the indicated time points, parasite burdens were determined as relative amounts of *Leishmania*-ITS1 to housekeeping m*GAPDH* by qPCR. **(A,C)**: average parasite burdens in IV and IP groups at each time point; **(B,D)**: individual parasite burden plots of IP and IV groups at the different time points; *P < 0.05; **P < 0.01; ****P < 0.0001.

After inoculation with *L. martiniquensis via* the intravenous route, the parasite burden in the liver gradually increased and reached a peak at 14 dpi. Subsequently, the parasitic load was reduced to the near baseline level at 28 dpi, and the infection was resolved at 112 dpi. Intraperitoneal infection revealed a significantly lower parasite burden in the liver than that of intravenous inoculation (Figures 3A,B). Although a weak positive correlation was observed, there was no significant correlation between the parasite burden and liver weight either after IV or IP infection [IV group: r_(16)_ = 0.46, r^2^ = 0.21, *P* = 0.07; IP group: r_(16)_ = 0.31, r^2^ = 0.10, *P* = 0.24] (Supplementary Tables 1–4).

In contrast, parasite replication was not evident in the early acute phase of *L. martiniquensis* infection (7 dpi). From 14 dpi, the parasite burden in the spleen began to increase, and it maintained this high parasitic load at 112 dpi. The course of infection in the spleen in the IP and IV groups were comparable, but the parasite burden in the spleen in the IV group was significantly higher than in the IP group (Figures 3C,D). Interestingly, the parasite burden and spleen weight after IV and IP infection showed a significantly moderate positive correlation [IV group: r_(16)_ = 0.54, r^2^ = 0.29, *P* = 0.03; IP group: r_(16)_ = 0.69, r^2^ = 0.47, *P* = 0.003] (Supplementary Tables 1–4).

### Histopathological Changes and Immune Responses to *L. martiniquensis* Infection

#### Efficient Formation of Liver Granulomas After *L. martiniquensis* Infection

**Figure 4:**
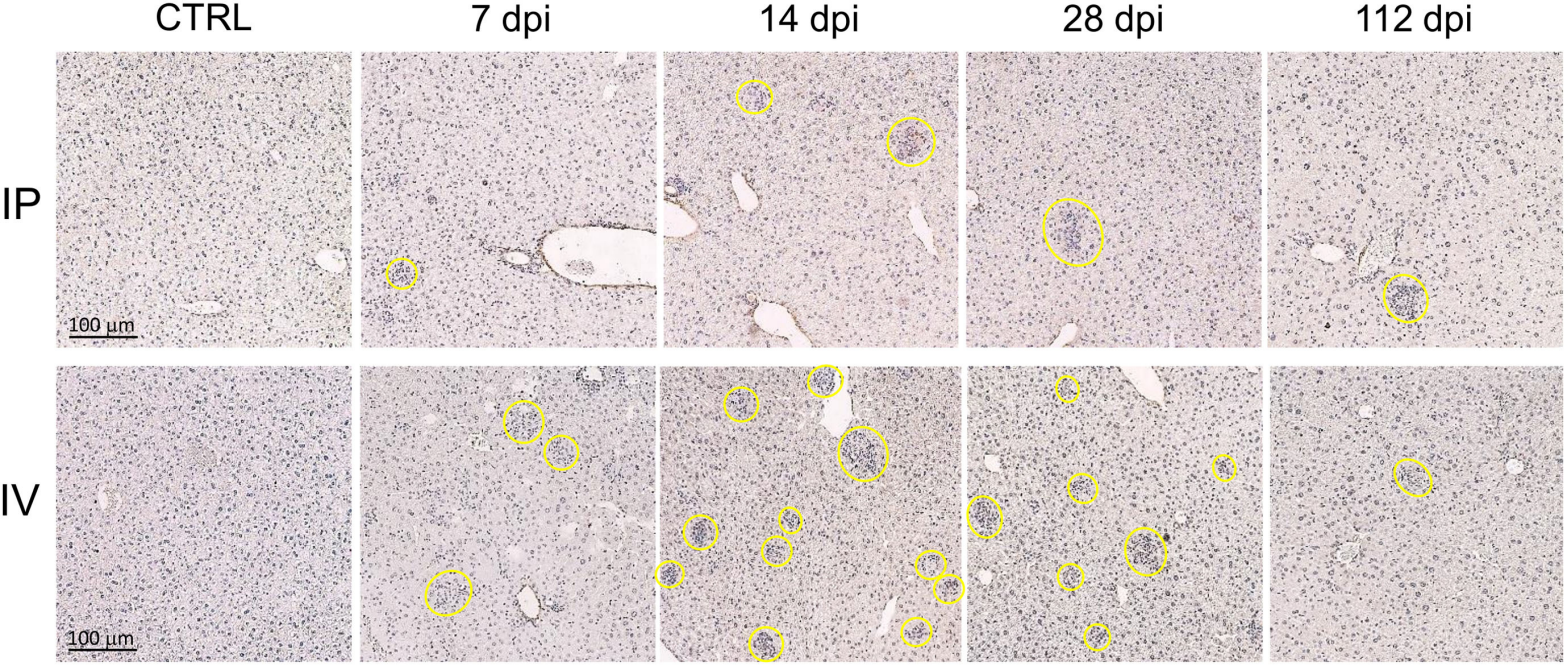
Hepatic granuloma formation denoting cell-mediated immune responses after *Leishmania martiniquensis* infection in BALB/c mice *via* intraperitoneal and intravenous routes. Representative granuloma lesions in liver sections are shown by immunostaining with anti-*L. martiniquensis* serum at 7, 14, 28, and 112 dpi. The yellow circles indicate mature granuloma surrounding the infected foci. Scale bar: 100 μm.

**Figure 5:**
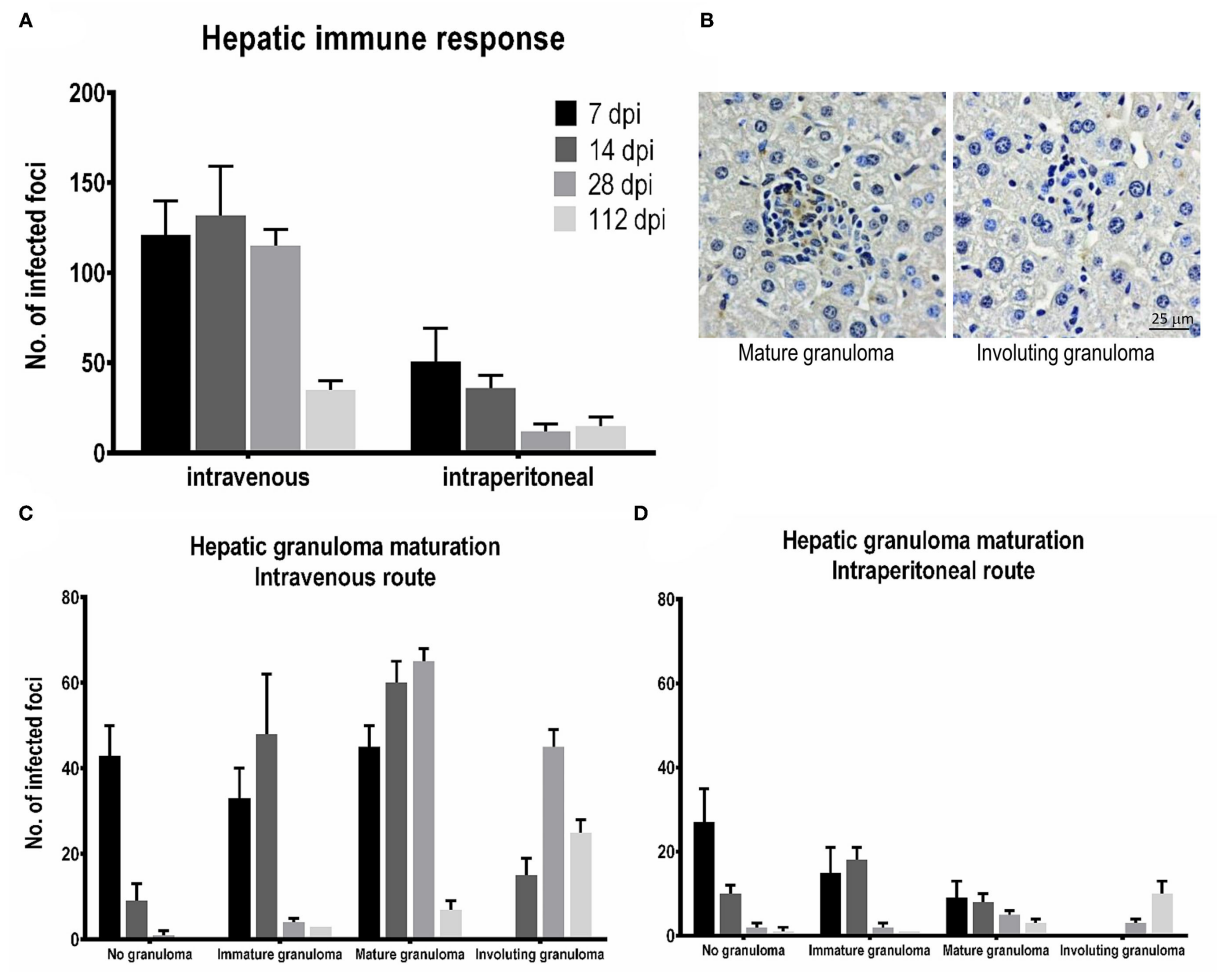
Tissue responses and hepatic granuloma maturation after *Leishmania martiniquensis* infection in BALB/c mice *via* intraperitoneal (IP) and intravenous (IV) routes. **(A)** The number of foci infected at different time points was counted from 25 consecutive microscopic fields. **(B)** Representative mature granulomas and involuting granulomas in the liver after infection with *L. martiniquensis*, immunostained with anti-*L. martiniquensis* serum. Kinetics of tissue responses in each category, including no granuloma, immature granuloma, mature granuloma, and involuting granuloma, at 7, 14, 28, and 112 dpi in the liver after *L. martiniquensis* infection *via* the IV route **(C)**, and IP route **(D)**.

The progression of liver granuloma formation, from immature granulomas (IG) to mature granulomas (MG), was demonstrated in both the IV and IP inoculation groups (Figure 4). The immunohistochemical analysis indicated the advancement of involuting granuloma, in which the amastigote disappeared with tissue healing (Figure 5B). There was a higher intensity of mature granulomas in the liver after IV infection than that after IP infection (Figure 4).

The total number of infected foci in the livers after IV infection reached a peak at 14 dpi and was drastically reduced at 112 dpi. The number of infected foci and the number of each granuloma after IP infection was lower than those after IV infection (Figures 5A,D), indicating the lower parasite burden. In the IV inoculation group, the number of IGs gradually increased from 7 to 14 dpi, while the MGs developed successively from 7 to 28 dpi. Involuting granulomas began to present at 14 dpi and remained up to 112 dpi (Figure 5C).

#### Disorganization of the Spleen With Parasitized Macrophages

**Figure 6:**
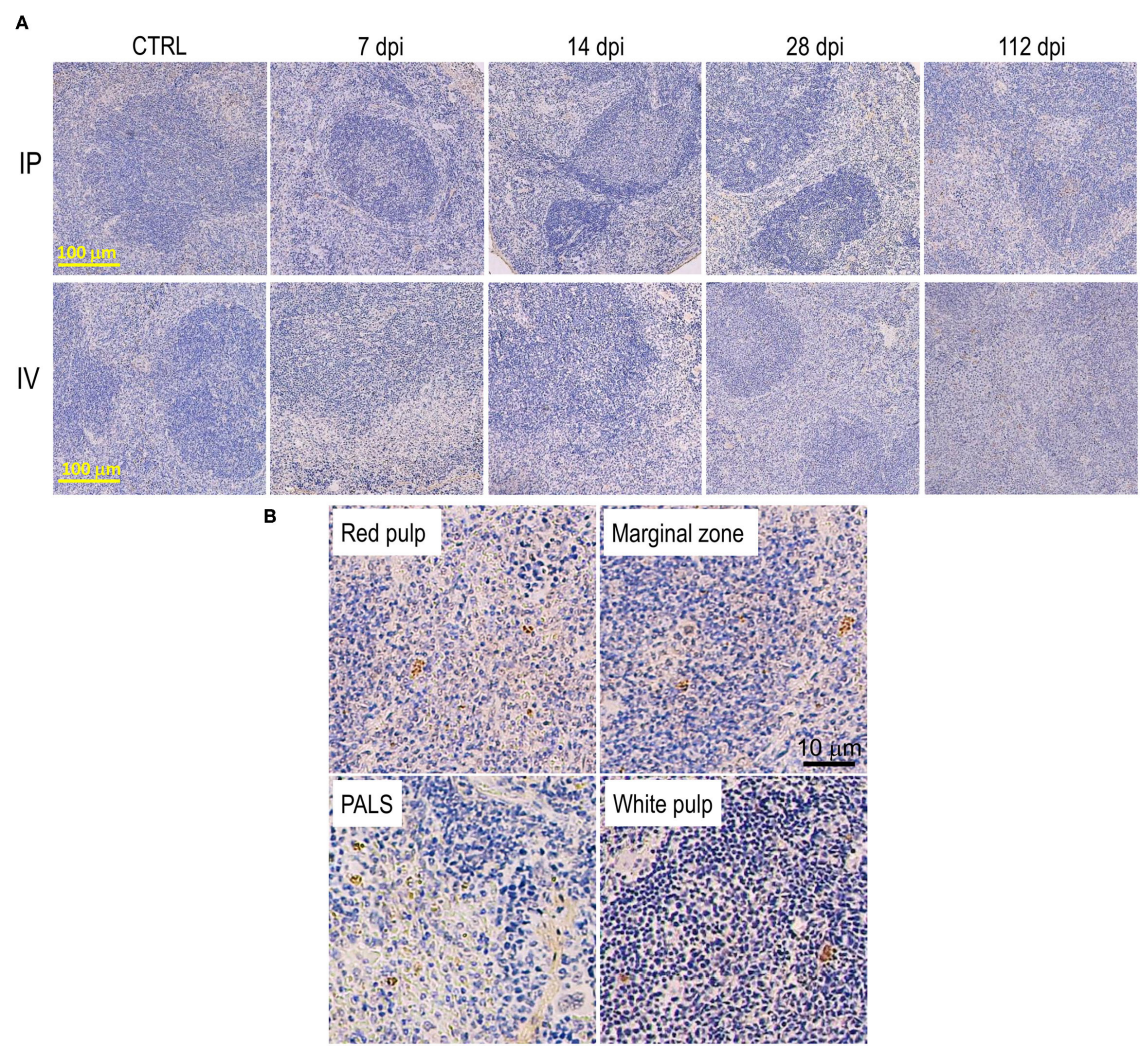
Disorganization of the spleen after *Leishmania martiniquensis* infection in BALB/c mice *via* intraperitoneal and intravenous routes. **(A)** Representative lesions of the spleen after infection, including white pulp disruption and red pulp expansion, which started at 28 dpi and was considerably present at 112 dpi. Scale bar: 100 μm. **(B)** Parasitized macrophages in the red pulp, marginal zone, periarteriolar lymphocyte sheath (PALS) and white pulp by immunostaining with anti-*L. martiniquensis* serum. Scale bar: 10 μm.

In the early stage of infection (7–14 dpi), no significant change in splenic structure was observed (Figure 6A). Some parasitized macrophages were observed in the marginal zone and in the red pulp (Figure 6B). However, structural changes of the spleen began from 28 dpi, with a reduction in size of white pulp along with the expansion of the red pulp area. At 112 dpi, the white pulp disorganized and the size was markedly reduced (Figure 6A), with several parasitized macrophages identified in several locations, including in the white pulp and the periarteriolar lymphocyte sheath (PALS) (Figure 6B). Histopathological changes in the spleen in the IP group were similar, but occurred later than in the IV group; however, the breakdown of the lymphoid follicles and the architecture of the white pulp of IP and IV mice at 112 dpi was comparable (Figure 6A). No granuloma formation was observed in the spleen after *L. martiniquensis* infection.

### Upregulation of Th1 Cytokines and Anti-leishmanial Molecule mRNA in the Liver and Spleen After Infection With *L. martiniquensis*

**Figure 7:**
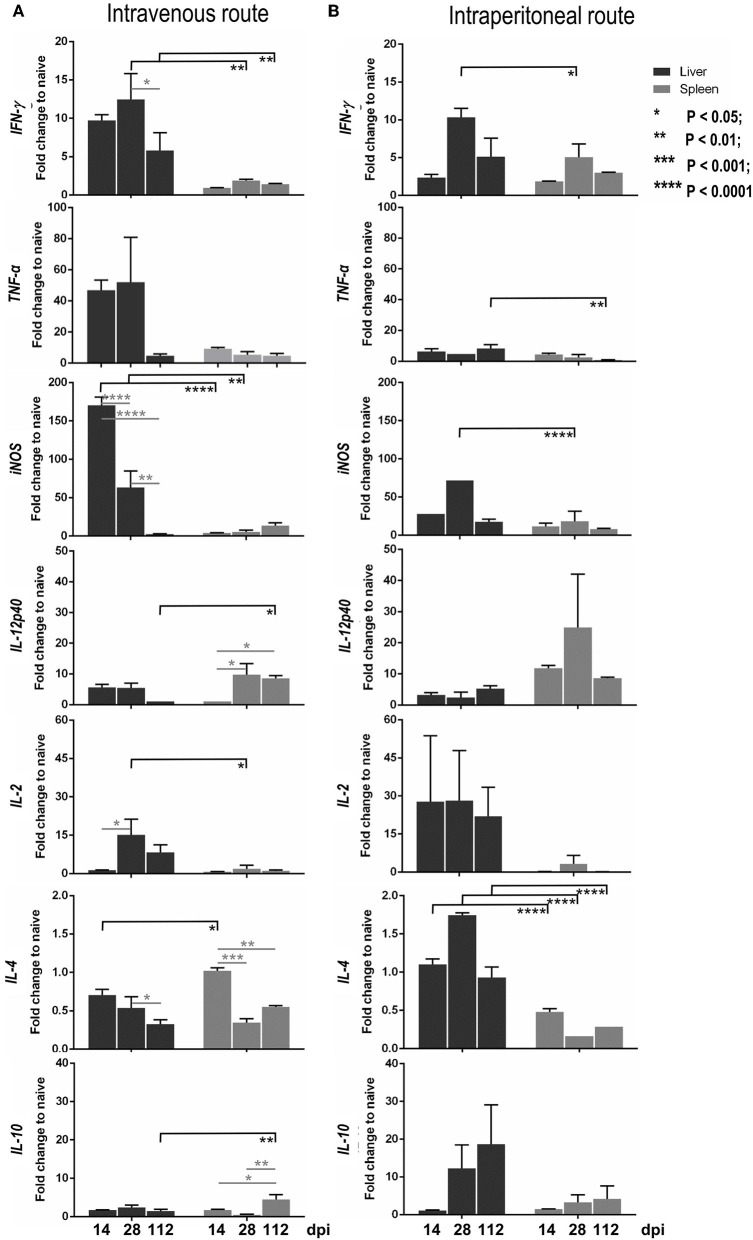
Kinetics of the expression levels of cytokine and *iNOS* mRNAs in the liver and spleen infected with *Leishmania martiniquensis* of BALB/c mice by intravenous **(A)** and intraperitoneal **(B)** routes, determined by RT-qPCR. The amounts of *IFN-*γ*, TNF-*α*, IL-12p40, IL-2, IL-4, IL-10*, and *iNOS* mRNA relative to housekeeping m*GAPDH* mRNA at 14, 28, and 112 dpi were calculated and presented as the fold change compared to levels in naïve mice.

In the intravenous inoculation group, the hepatic mRNA level expression of *IFN-*γ*, TNF-*α*, IL-12p40, IL-2*, and *iNOS* progressively increased from 14 to 28 dpi, and were higher than those of naïve mice (Figure 7A; black bar). Subsequently, these Th1 cytokines and antileishmanial molecules decreased at 112 dpi when the parasite burden in the liver was resolved. The release of the suppressive cytokine, *IL-10*, and Th2 cytokine, *IL-4*, were not induced (less than a one-fold change compared to the level of naïve mice; Figure 7A). After intraperitoneal infection, lower expressions *of IFN-*γ*, TNF-*α*, IL-12p40*, and *iNOS* were noticeable compared to the IV route group, but this was not the case for *IL-2, IL-4*, and *IL-10* (Figure 7B; black bar).

In the spleen, after IV and IP infections, the levels of Th1 cytokines, including *IFN-*γ*, TNF-*α*, iNOS*, and *IL-2*, were considerably lower than those in the liver (Figure 7, gray bar), relevant to the uncontrolled parasite burdens in the spleen. Interestingly, the level of *IL-12p40* in the spleen, *via* either IV or IP inoculation, was significantly higher than that in the liver. In addition, a high transcription level of *IL-10* in the spleen was observed at 112 dpi after infection *via* the IV route and was significantly higher than that in the liver (Figure 7A).

The Pearson correlation coefficient calculation between the parasite burden and the cytokine level in the liver and spleen after IV or IP infections revealed that there was a significantly strong and moderate positive correlation between the parasite burden and *iNOS* expression level in the liver and spleen after IV infection, respectively [liver: r_(12)_ = 0.78, r^2^ = 0.60, *P* = 0.003; spleen: r_(12)_ = 0.63, r^2^ = 0.39, *P* = 0.03]. In addition, the parasite burden, and the level of *IL-10* in the spleen after IV infection showed a significantly moderate positive correlation: r_(12)_ = 0.72, r^2^ = 0.51, *P* = 0.009. Although there was no significant changes in the level of *TNF-*α, its expression was negatively correlated with the parasite burden in the spleen after either inoculation by IV route [r_(12)_ = −0.30, r^2^ = 0.09, *P* = 0.33] or by the IP route: r_(12)_ = −0.33, r^2^ = 0.11, *P* = 0.29 (Supplementary Tables 1–4).

## Discussion

In the present study, the organ-specific immune responses of *L. martiniquensis* infection in the liver and spleen were investigated using the inbred BALB/c mouse strain as the animal model. After intravenous and intraperitoneal inoculation with 5 × 10^6^ promastigotes of *L. martiniquensis*, strain MHOM/TH/2011/PG, infection outcomes, including parasitic burdens, histopathological lesions, and Th1/Th2 immune responses, were evaluated during the early phase (28 dpi) and the chronic phase (up to 112 dpi). The resolving infection in the liver together with the efficient evolution of hepatic granuloma formation were demonstrated in both routes of inoculation, although the intraperitoneal route allowed poorer assessment of outcomes. *Leishmania martiniquensis* persistence with splenic disorganization and disruption was substantially observed. A notable moderate positive correlation of splenic weight and parasitic burden after IV and IP infections was observed. This study highlighted the upregulation of mRNA transcription levels of Th1 cytokines, including *IFN-*γ*, TNF-*α*, IL-12p40*, and *iNOS*, in the liver and spleen, although the levels in the spleen were significantly lower. Interestingly, a high expression of *IL-10* was observed in the spleen during the chronic phase, which had a significantly moderate correlation with parasitic persistence. Furthermore, in the reciprocal study, *L. martiniquensis* DNA was detected in the buffy coat, bone marrow, salivary gland, and kidney samples using conventional PCR (29).

Hepatomegaly was observed after IP and IV infection in the early phase (up to 28 dpi) during the proliferation of the parasite, and the development of liver granulomas was observed. However, the infection resolved and the amastigotes finally disappeared. The infection pattern and parasitic load in BALB/c mice in this study mimicked other experimental VL infections with *L. donovani* (9, 10, 22, 26, 30, 31) and *L. infantum* (32, 33) in BALB/c or C57BL/6 mice that present a more susceptible mouse genetic background in VL outcomes (11). Efficient granuloma development surrounding infected Kupffer cells is the key event in the control of hepatic infection (10, 22, 24, 34). Immunohistochemical analysis confirmed the existence of efficient mature granulomas and involuting granulomas, indicating that *L. martiniquensis*-infected BALB/c mice could generate liver cell-mediated immunity.

Various chemokines, cytokines, and *iNOS* have been shown to affect the development of hepatic granulomas in VL (10, 22, 24). Following *L. donovani* infection, the chemokines *IFN-*γ *inducible protein-10* and *monocyte chemoattractant protein-1* were secreted by Kupffer cells, monocytes, and T cells in both a T cell-independent and dependent manner, resulting in the recruitment of monocytes and CD4^+^ and CD8^+^ T cells to the developing granulomas (26). Th1 cytokines, such as TNF-α and IFN-γ, are required for the stimulation of chemokine production and the generation of leishmanicidal molecules (reactive nitrogen intermediates that are induced by infected Kupffer cells). High transcription levels of hepatic *IFN-*γ and *TNF-*α mRNA with extraordinary *iNOS* mRNA expression after intravenous *L. martiniquensis* infection was correlated with the evolution of effective tissue responses, resulting in the successful killing of parasites in the liver (9, 10, 22, 26).

The major Th2 cytokine, IL-4, secreted by CD4^**+**^ T cells also contributes to the development of hepatic granulomas (35). However, in the present study, the transcriptional level of *IL-4* mRNA was not increased, probably due to the downregulation of micro-RNAs, which could limit the differentiation of naïve CD4^+^ T cells to Th2 cells, as observed in *L. donovani* infection (36, 37). Expression of IL-10, an important immunoregulatory Th2 cytokine, was induced in the liver after *L. martiniquensis* infection in the early stage (0–12 dpi), rather than in the later stage, of *L. donovani* infection (38). Another study on *IL-10*-deficient BALB/c mouse models showed that *IL-10*^**−/−**^ mice were resistant to *L. donovani* infection of VL (39, 40) with upregulation of *IFN-*γ and *iNOS* mRNA expression. As yet, there is no information to suggest that IL-4 and IL-10 are engaged in downregulating the Th1-type immune response in human leishmaniasis caused by *L. donovani* (41); however, further study is needed to identify the role of Th2 cells in *L. martiniquensis* infection in both human and murine models.

In contrast to the liver, this study revealed the parasite persistence was associated with splenomegaly after *L. martiniquensis* infection *via* either the intravenous or intraperitoneal route. The parasitic burden slowly increased from 7 dpi but was uncontrolled at 112 dpi. The progressive breakdown of the splenic architecture and lymphoid depletion in the white pulps was demonstrated with high levels of *L. martiniquensis* amastigotes without granuloma formation. Due to the interaction between the spleen and the blood system, the spleen is an important site for the generation of immunity against viscerotropic *Leishmania* infection, and it is a critical site in the acute phase for the generation of immune responses and disease resolution in the liver (26). The presence of parasitized macrophages in many locations after *L. martiniquensis* infection in this study was relevant to the experimental studies of *L. donovani* and *L. infantum*, which resulted in the rapid removal of amastigotes from the circulation to the spleen by macrophages and dendritic cells of the splenic marginal zone, red pulps, and PALS (26).

In the present study, significant levels of splenic *IL-12p40* mRNA were observed at 28 dpi and 112 dpi *via* both IV and IP routes, compared to the initial stage at 7 dpi after *L. martiniquensis* infection. The increase in *IL-12p40* mRNA subunit expression from dendritic cells, but not macrophages, after *L. donovani* infection in BALB/c mice was an early rapid and transient burst (42, 43). IL-12 is critical for the activation of host-protective CD4^+^ T cells, and potentially CD8^+^ T cells into IFN-γ-producing T cells, and thus, IFN-γ is essential for the activation of macrophages and the elimination of parasites (44). High transcription levels of splenic *TNF-*α mRNA at 14 and 28 dpi were demonstrated, although a negative correlation with the parasitic burden was observed. After experimental *L. donovani* infection in BALB/c mice, TNF is a common primary mediator of splenic pathology for the loss of both marginal zone (45) and stromal cells in the PALS (46). Stanley and Engwerda (26) determined that TNF production was an essential characteristic of the effective response of the liver to viscerotropic *Leishmania* infection. However, excessive *TNF-*α production in the splenic lymphoid environment is responsible for the architectural damage and immunological dysfunction associated with this chronic inflammatory state. Finally, in the present study with *L. martiniquensis* infection, a significant correlation of *IL-10* levels with the parasitic load in the chronic phase at 112 dpi was noted and was probably associated with the spike in *TNF-*α mRNA levels (26). The elevated levels of *TNF-*α in the spleen were associated with the induction of IL-10 expression, which significantly contributed to the establishment of infection (39).

Variation in disease outcomes in experimental murine studies is commonly influenced by parasite strain and virulence, the route of inoculation, and the dose of the parasite (11, 12). In the present study, the *Leishmania martiniquensis* strain MHOM/TH/2011/PG was used for infection, and this parasite was derived from an immunocompetent VL patient, who was negative for HIV/AIDs coinfection (1, 16). Although this strain was the same zymodeme of MON-229 as the MHOM/MQ/92/MAR1 strain (15), and was previously employed for experimental studies using the BALB/c mouse model by Garin et al. (13), the disease outcome was somewhat different. The MHOM/MQ/92/MAR1 strain proliferated in the liver, but the parasite clearance extended for a longer period, almost 150 dpi. This was probably due to the different virulence of the MHOM/MQ/92/MARl strain that originated from an immunosuppressed HIV patient (15). Regarding the route of infection, an unexpectedly lower parasitic burden was demonstrated in the intraperitoneal group compared to the intravenous group in the present study. Although the same inoculation route was used, the disease outcome after IP inoculation in the present study was incompatible with the study of Intakhan et al. (14), in which an inability to destroy the parasite in the liver of BALB/c mice without hepatomegaly was curiously shown, up to 16 weeks post-infection. This was probably influenced by: (1) the more virulent *L. martiniquensis* strain [the MHOM/TH/2013/LSCM3 strain that derived from DCL/VL with an HIV co-infection (5)] and (2) the higher inoculum dose (2 × 10^7^ promastigotes per mouse), which was two times higher than the dosage used in the present study.

Lastly, our established qPCR in the present study, using L.ITS1.PCM2.4.6 primers, could differentiate *L. martiniquensis* (Tm = 84.9°C) from *L. orientalis* (Tm = 81.0°C), two major species endemic in Thailand (3), and from *L. infantum* (Tm = 82.6°C), a species of sporadic occurrence (47). In the last two decades, the RT-qPCR application for the detection, quantification and genotyping of *Leishmania* species represents an advanced automation technique with high throughput, rapidity, and high sensitivity (48, 49). qPCR has been used to quantify *Leishmania* loads in target organs of experimental animal models (23, 50, 51). In a different approach, using primers common for different species, differences in Tm of the amplified PCR amplicons were exploited to discriminate the *Leishmania* species. Nicolas et al. (52) optimized an assay to distinguish the Old World species *L. major, L. donovani, L. tropica*, and *L. infantum* targeting minicircle kinetoplast DNA. Consequently, de Morais et al. (53) identified two Tm ranges to differentiate between two groups of species that cause CL in Brazil, in which group 1 included *L*. (*Viannia) braziliensis, L*. (*V*.) *panamensis, L*. (*V*.) *lainsoni, L*. (*V*.) *guyanensis*, and *L*. (*V*.) *shawi* (Tm = 78–79.99°C); and group 2 included *L*. (*V*.) *naiffi, L*. (*Leishmania*) *amazonensis*, and *L*. (*L*.) *mexicana* (Tm = 80–82.2°C). Therefore, a field application of our established qPCR for epidemiological work should be prospectively extended in the countries where different viscerotropic *Leishmania* species, including *L. (M.) martiniquensis, L. (M.) orientalis*, and *L. (L.) donovani* species complex coexist.

## Conclusions

The present study was an alternative experimental study of *L. martiniquensis* infection in BALB/c mice that revealed visceral dissemination of the parasite, histopathological alteration, and Th1/Th2 immune responses in two main target organs: the liver and the spleen. This model should be employed for further study of pathogenesis in immunocompromised murine models, the mechanism of parasite persistence, and the development of effective therapeutic and prophylactic strategies.

## Data Availability Statement

The raw data supporting the conclusions of this article will be made available by the authors, without undue reservation.

## Ethics Statement

The animal study was reviewed and approved by National Institute of Animal Health Animal Use Committee (EA-001/57(R).

## Author Contributions

ST and WS: conceptualization, data curation, research funding acquisition, project administration, draft revision, and author proof revision. WS, TK, NS, MJ, JW, MK, and ST: methodology. WS, TK, and ST: validation. WS, TK, NS, MJ, JW, and ST: investigation. WS, MJ, SL, and ST: resources. WS, NS, and ST: visualization and formal analysis. SL: supervision. ST: publication fee acquisition, writing—original draft preparation, and software. WS and SL: original draft editing. All authors have read and agreed to the published version of the manuscript.

## Funding

This study was supported by the Research Center of Producing and Development of Products and Innovations for Animal Health and Production, Faculty of Veterinary Medicine, Chiang Mai University (FVM-CMU); the FVM-CMU Research Fund Y2016 (Grant Number: R000016307) for ST; the Thailand Research Fund and Chulalongkorn University (Grant Number: MRG5680172); Chulalongkorn University-Veterinary Science Research Fund (fiscal year 2012) and Special Project Research fund (academic year 2014); Faculty of Veterinary Science, Chulalongkorn University for WS; and the 90^th^ Anniversary of Chulalongkorn University Fund (Ratchadaphiseksomphot Endowment Fund, fiscal year 2017) for NS.

## Conflict of Interest

The authors declare that the research was conducted in the absence of any commercial or financial relationships that could be construed as a potential conflict of interest.

## Publisher's Note

All claims expressed in this article are solely those of the authors and do not necessarily represent those of their affiliated organizations, or those of the publisher, the editors and the reviewers. Any product that may be evaluated in this article, or claim that may be made by its manufacturer, is not guaranteed or endorsed by the publisher.
