# Supplementary material for: Liver- and Spleen-Specific Immune Responses in Experimental Leishmania martiniquensis Infection in BALB/c Mice
Source: Front Vet Sci. 2021 Dec 17;8:794024. doi: 10.3389/fvets.2021.794024 (PMC8718515; doi:10.3389/fvets.2021.794024)
Supplement: Supplementary file 1 [file Data_Sheet_1.PDF]

Article

Liver and Spleen Specific Immune Responses in Experimental *Leishmania martiniquensis* Infection in BALB/c Mice

Table S1. Raw Data and the Pearson correlation (r) analysis of the parasite burden in the liver (*Leishmania*-ITS1/1000x m*GAPDH*) and the liver weight (mg) at 7-, 14-, 28-, and 112 dpi, or the mRNA transcription levels of cytokines and iNOS in the liver (per 1000x m*GAPDH*) after *L. martiniquensis* infection via intravenous route.

Parameter in **Liver**	*L. martiniquensis* infection via **Intravenous route**																r*	*p***	N
	7 dpi				14 dpi				28 dpi				112 dpi						
	mice1	mice2	mice3	mice4	mice1	mice2	mice3	mice4	mice1	mice2	mice3	mice4	mice1	mice2	mice3	mice4			
Parasite burdens	0.312	0.018	0.005	0.002	6.489	4.359	1.119	6.456	0.868	0.549	0.308	1.256	0.002	0.005	0.004	0.003	1.000	-	16
Weight (mg)	1.46	1.58	1.53	1.56	1.57	1.61	1.79	1.80	1.57	1.61	1.52	1.65	1.58	1.58	1.36	1.58	0.461	0.07	16
IFN-g	n/a	n/a	n/a	n/a	3.922	5.759	4.694	4.598	3.748	7.703	2.977	9.890	1.895	0.572	2.972	5.941	0.126	0.70	12
TNF-a	n/a	n/a	n/a	n/a	7.090	6.975	3.706	5.227	4.269	1.347	16.845	3.104	0.846	0.650	0.626	0.225	0.289	0.36	12
iNOS	n/a	n/a	n/a	n/a	20.077	15.145	17.166	15.695	6.337	3.240	12.432	3.368	0.191	0.411	0.321	0.141	**0.777**	**.003**	**12**
IL-12p40	n/a	n/a	n/a	n/a	0.364	0.835	0.469	0.573	0.300	n/a	0.800	n/a	n/a	n/a	n/a	n/a	-0.057	0.91	6
IL-2	n/a	n/a	n/a	n/a	0.014	0.010	0.011	0.013	0.046	0.050	0.184	0.274	0.150	0.074	0.058	0.018	-0.394	0.21	12
IL-4	n/a	n/a	n/a	n/a	0.944	1.249	0.756	0.399	0.684	0.579	1.313	0.638	0.267	0.522	0.379	n/a	0.237	0.48	11
IL-10	n/a	n/a	n/a	n/a	0.653	0.525	0.531	0.577	0.665	0.459	0.707	1.419	0.925	0.317	0.412	0.253	0.047	0.89	12

* Strength of relationship: < 0.3 = None or very weak; 0.31-0.5: weak; 0.51-0.7: moderate; > 0.7: strong.

** Correlation is significant when $p < 0.05$ level.

N: number of values; n/a: not applicable (due to inadequate mRNA and cDNA template.
