# Supplementary material for: Liver- and Spleen-Specific Immune Responses in Experimental Leishmania martiniquensis Infection in BALB/c Mice
Source: Front Vet Sci. 2021 Dec 17;8:794024. doi: 10.3389/fvets.2021.794024 (PMC8718515; doi:10.3389/fvets.2021.794024)
Supplement: Supplementary file 2 [file Data_Sheet_2.PDF]

Article

Liver and Spleen Specific Immune Responses in Experimental *Leishmania martiniquensis* Infection in BALB/c Mice

Table S2. Raw Data and the Pearson correlation (r) analysis of the parasite burden in the liver (*Leishmania*-ITS1/1000x m*GAPDH*) and the liver weight (mg) at 7-, 14-, 28-, and 112 dpi, or the mRNA transcription levels of cytokines and iNOS in the liver (per 1000x m*GAPDH*) after *L. martiniquensis* infection via intraperitoneal route.

Parameter in **Liver**	*L. martiniquensis* infection via **Intraperitoneal route**																r*	*p***	N
	7 dpi				14 dpi				28 dpi				112 dpi						
	mice1	mice2	mice3	mice4	mice1	mice2	mice3	mice4	mice1	mice2	mice3	mice4	mice1	mice2	mice3	mice4			
Parasite burdens	0.456	0.003	0.000	0.002	0.231	0.148	0.111	0.067	0.015	0.028	0.113	0.002	0.004	0.010	0.001	0.001	1.000	-	16
Weight (mg)	1.66	1.52	1.33	1.4	1.51	1.58	1.32	1.23	1.41	1.38	1.44	1.15	1.73	1.6	1.33	1.66	0.313	0.24	16
IFN-g	n/a	n/a	n/a	n/a	2.872	2.600	2.357	1.233	8.823	7.679	9.799	12.930	7.656	9.936	0.710	1.177	-0.322	0.31	12
TNF-a	n/a	n/a	n/a	n/a	1.415	1.150	2.696	0.819	n/a	n/a	n/a	1.110	0.792	1.246	2.222	3.461	-0.126	0.75	9
iNOS	n/a	n/a	n/a	n/a	2.814	n/a	n/a	n/a	n/a	7.183	n/a	1.800	n/a	n/a	1.277	2.323	0.051	0.94	5
IL-12p40	n/a	n/a	n/a	n/a	0.474	0.700	0.905	0.255	0.100	0.100	n/a	1.300	0.700	1.300	n/a	n/a	-0.156	0.69	9
IL-2	n/a	n/a	n/a	n/a	0.965	0.015	0.018	0.014	0.027	n/a	0.012	0.782	0.019	0.020	0.377	0.387	0.237	0.48	11
IL-4	n/a	n/a	n/a	n/a	0.991	0.914	0.722	n/a	1.471	n/a	1.374	n/a	0.674	0.667	1.089	n/a	0.037	0.93	8
IL-10	n/a	n/a	n/a	n/a	0.395	0.363	0.492	0.330	10.599	n/a	2.306	0.905	1.271	1.281	6.865	16.772	-0.469	0.15	11

* Strength of relationship: < 0.3 = None or very weak; 0.31-0.5: weak; 0.51-0.7: moderate; > 0.7: strong.

** Correlation is significant when $p < 0.05$ level.

N: number of values; n/a: not applicable (due to inadequate mRNA and cDNA template.
