# Supplementary material for: Liver- and Spleen-Specific Immune Responses in Experimental Leishmania martiniquensis Infection in BALB/c Mice
Source: Front Vet Sci. 2021 Dec 17;8:794024. doi: 10.3389/fvets.2021.794024 (PMC8718515; doi:10.3389/fvets.2021.794024)
Supplement: Supplementary file 4 [file Data_Sheet_4.PDF]

Article

Liver and Spleen Specific Immune Responses in Experimental *Leishmania martiniquensis* Infection in BALB/c Mice

Table S4. Raw Data and the Pearson correlation (r) analysis of the parasite burden in the spleen (*Leishmania*-ITS1/1000x m*GAPDH*) and the spleen weight (mg) at 7-, 14-, 28-, and 112 dpi, or the mRNA transcription levels of cytokines and *iNOS* in the spleen (per 1000x m*GAPDH*) after *L. martiniquensis* infection via intraperitoneal route.

Parameter in **Spleen**	*L. martiniquensis* infection via **Intraperitoneal route**																r*	*p***	N
	7 dpi				14 dpi				28 dpi				112 dpi						
	mice1	mice2	mice3	mice4	mice1	mice2	mice3	mice4	mice1	mice2	mice3	mice4	mice1	mice2	mice3	mice4			
Parasite burdens	0.000	0.000	0.000	0.000	0.504	0.338	0.526	0.155	0.190	0.882	1.582	0.005	3.029	6.878	4.689	0.326	1.000	-	16
Weight (mg)	0.19	0.15	0.13	0.2	0.13	0.09	0.11	0.1	0.11	0.12	0.14	0.09	0.21	0.25	0.2	0.18	0.461	0.072	16
IFN-g	n/a	n/a	n/a	n/a	5.172	5.191	4.910	5.212	9.554	27.995	8.763	9.018	8.146	7.814	8.463	8.610	-0.028	0.93	12
TNF-a	n/a	n/a	n/a	n/a	4.712	6.846	10.873	6.947	0.648	13.736	1.137	1.348	1.920	1.719	1.207	1.663	-0.333	0.29	12
iNOS	n/a	n/a	n/a	n/a	0.686	0.793	2.422	0.845	0.409	5.700	0.680	0.629	0.748	1.080	0.589	0.800	-0.100	0.76	12
IL-12p40	n/a	n/a	n/a	n/a	1.034	1.338	n/a	n/a	0.679	7.652	0.985	0.658	0.819	0.950	0.813	0.000	-0.115	0.75	10
IL-2	n/a	n/a	n/a	n/a	0.101	0.128	0.173	0.171	0.024	6.977	0.018	0.033	0.083	0.052	0.052	0.141	-0.113	0.73	12
IL-4	n/a	n/a	n/a	n/a	1.455	0.978	1.080	1.173	n/a	n/a	0.395	n/a	n/a	0.702	n/a	n/a	-0.487	0.13	6
IL-10	n/a	n/a	n/a	n/a	2.551	2.942	2.726	2.413	n/a	16.128	0.665	1.135	1.339	1.545	1.376	25.883	-0.288	0.39	11

* Strength of relationship: < 0.3 = None or very weak; 0.31-0.5: weak; 0.51-0.7: moderate; > 0.7: strong.
** Correlation is significant when $p < 0.05$ level.
N: number of values; n/a: not applicable (due to inadequate mRNA and cDNA template.
