# Supplementary figures and images for: Liver- and Spleen-Specific Immune Responses in Experimental Leishmania martiniquensis Infection in BALB/c Mice
Source: Front Vet Sci. 2021 Dec 17;8:794024. doi: 10.3389/fvets.2021.794024 (PMC8718515; doi:10.3389/fvets.2021.794024)

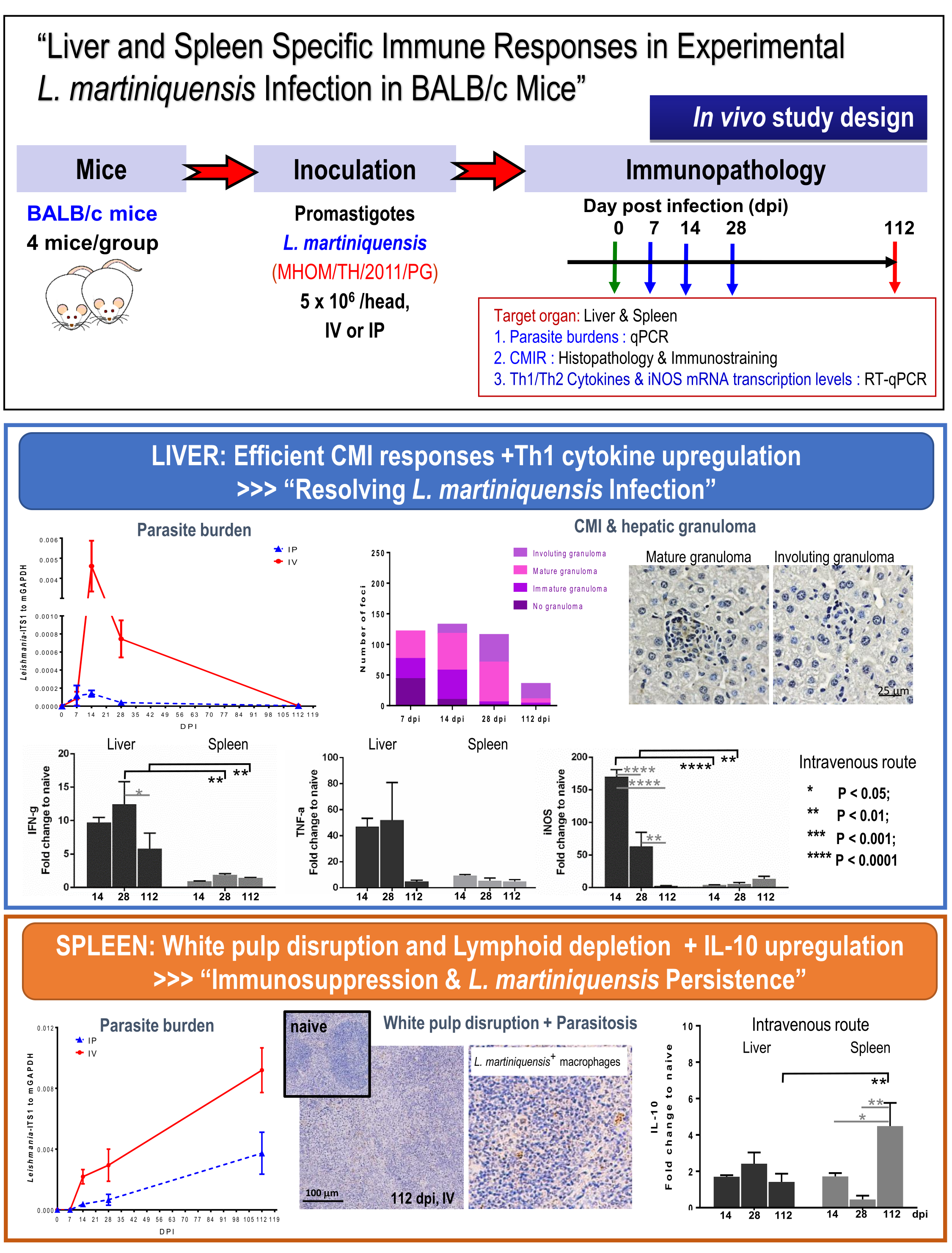

Supplement: Supplementary file 5 [file Image_1.TIFF]
